# High Prevalence and Genetic Heterogeneity of *Anaplasma marginale* in Smallholder Bovine Populations of Pakistan, and Its Implications

**DOI:** 10.3390/pathogens14050499

**Published:** 2025-05-20

**Authors:** Abdul Ghafar, Waseem Shaukat, Muhammad Waqas, Charles G. Gauci, Robin B. Gasser, Abdul Jabbar

**Affiliations:** 1 Melbourne Veterinary School, The University of Melbourne, Werribee, VIC 3030, Australia; charlesg@unimelb.edu.au (C.G.G.); robinbg@unimelb.edu.au (R.B.G.); 2 Faculty of Veterinary Medicine, University of Calgary, Calgary, AB T2N 1N4, Canada; waseem.shaukat@ucalgary.ca; 3 Faculty of Organic Agricultural Sciences, University of Kassel, 34109 Kassel, Germany; waqasdairy2017@gmail.com; 4 Faculty of Nutritional, Food and Consumer Sciences, Fulda University of Applied Sciences, 36037 Fulda, Germany

**Keywords:** *Anaplasma marginale*, anaplasmosis, bovine, cattle, endemic stability, major surface protein B gene, nested PCR, Pakistan, smallholder farms, tick-borne pathogen, water buffalo

## Abstract

Bovine anaplasmosis, caused by *Anaplasma marginale*, is a major tick-borne disease in tropical and subtropical regions of the world, leading to significant production losses. Prolonged convalescence periods are common and surviving animals often become subclinical carriers. This study aimed to detect and characterise A. marginale in bovines in smallholder dairy farms across diverse climatic zones of Pakistan using molecular methods. In total, 321 blood DNA samples from apparently healthy cattle (n = 174) and buffaloes (n = 147) from six districts in Pakistan were tested for A. marginale using a nested PCR assay, targeting part of the major surface protein B gene (msp1β) as a genetic marker, followed by agarose gel electrophoresis and selective sequencing of amplicons from test-positive samples. Of the 321 DNA samples tested, 135 (42.1%) were test-positive for A. marginale. Prevalence was significantly higher in cattle (64.4%; 112/174) than in buffaloes (15.6%; 23/147), and female bovines (43.5%; 108/248) were more frequently infected than males (37%; 27/73). Phylogenetic analysis of the msp1β sequence data (n = 42) revealed that A. marginale from Pakistan clustered with those from Brazil, Thailand, South Africa, and the USA. This study represents the first comprehensive investigation of A. marginale from bovines from diverse agroecological zones of Pakistan and will further stimulate population genetic studies of A. marginale and investigations into the economic impact of subclinical infections in bovines in smallholder farming systems.

## 1. Introduction

Anaplasmosis, also known as gall sickness or tick fever, is one of the most economically significant tick-borne diseases (TBDs) of bovines in tropical and subtropical regions of the world [1,2]. It is caused by the obligate intracellular bacterium *Anaplasma* (*A*.) *marginale* (identified and named by Dr A. Theiler in 1910). It can be transmitted by at least 20 different tick species, primarily belonging to three genera: *Rhipicephalus*, *Ixodes* and *Dermacentor* [1,3,4]. Moreover, iatrogenic transmission also occurs for various strains of *A. marginale*, mainly through blood-contaminated fomites and dipteran flies [3]. Notably, both interstadial and intrastadial transmission pathways play a critical role in the persistence and spread of *A. marginale*. Although traditionally considered absent, recent evidence suggests that transovarial transmission of *A. marginale* can occur and male ticks can act as reservoirs of infection [3,5].

The clinical manifestation of *A. marginale* infection is more common in adult cattle (>2 years of age) and is characterised by high fever, anorexia, anaemia, weight loss, abortion in pregnant animals, production losses, and mortality in some cases [2,6]. Cattle are primarily affected by clinical anaplasmosis. However, several ruminant species (e.g., water buffalo, American bison, and deer) can acquire clinical infections [7] and surviving animals often develop a (subclinical) carrier state with life-long immunity [3]. However, these animals become reservoirs of infection for other animals and pose a risk to livestock health and productivity [2,3,8]. The economic losses caused by such infections are particularly significant in smallholder farming communities in countries such as Pakistan, where resources for disease diagnosis and control are scarce, and ticks and TBDs are highly prevalent [8,9,10,11,12].

Pakistan’s livestock sector contributes significantly to the food security and livelihoods of millions of smallholder farmers [13]. However, the high burden of TBDs, including anaplasmosis, poses challenges to the sustainability of bovine production in this country [8]. Previous studies have reported variable prevalence of *A. marginale* (2.0–53.7%), likely influenced by agroclimatic differences, tick distribution, and management practices [8]. The reliance on conventional diagnostic methods (such as Giemsa-stained blood smears and serological assays), limits accurate surveillance, particularly for detecting subclinical carrier animals [1,3,8]. Therefore, despite its widespread occurrence and associated losses, the molecular epidemiology of *A. marginale* remains poorly understood in Pakistan [8,14].

Molecular techniques, such as PCR-based assays, have revolutionised pathogen detection, and a nested PCR, targeting the part of major surface protein B gene (*msp1β*) gene, has shown greater sensitivity and specificity for the detection of *A. marginale*, particularly in animals with low parasitaemia [15]. However, while molecular tools such as PCR have been used in previous studies to detect *A. marginale* in Pakistan, there is still limited information on the genetic diversity of circulating strains in bovine populations. Therefore, the aim of this study was to apply a sensitive nested PCR-based and sequencing approach to detect and genetically characterise *A. marginale* in apparently healthy bovines from diverse agroecological zones of Pakistan. The findings provide valuable insights into the epidemiology of subclinical *Anaplasma* infection in smallholder farming systems. The implementation of this molecular tool is expected to contribute to improved disease surveillance and management of anaplasmosis in resource-poor farming communities.

## 2. Materials and Methods

**Figure 1:**
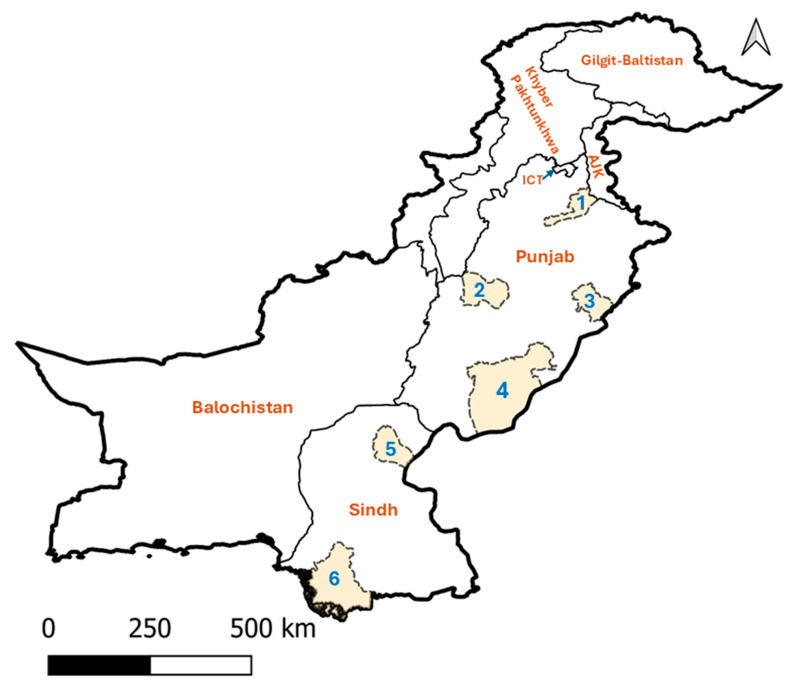
Map of Pakistan showing study areas (highlighted and numbered 1–6) included in this study. These districts are Jhelum (1), Okara (2), Layyah (3), Bahawalpur (4), Sukkur (5), and Thatta (6). Abbreviations: AJK, Azad Jammu and Kashmir; ICT, Islamabad Capital Territory.

Individual bovine blood DNA samples tested in this study were collected from apparently healthy cattle and water buffaloes (*Bubalus bubalis*) as part of a previously published study [16]. During the same sampling period, each animal was also examined for tick infestation through visual inspection and manual palpation of predilection sites, following the protocol described previously [11]. The ethics approval for bovine blood sampling was obtained from the Animal Ethics Committee at the University of Melbourne (permit no. 714216). A total of 321 DNA samples was available for this study, covering 149 smallholder farms (having ≤10 bovines) across 30 villages in two provinces: Punjab (districts: Bahawalpur, Jhelum, Layyah, Okara) and Sindh (districts: Sukkur, Thatta) (Figure 1, Table 1). These districts represent five distinct agroecological zones of Pakistan, where much of the national bovine population is inhabited. Blood was collected via jugular venipuncture into EDTA-coated vacutainers (BD, Franklin Lakes, NJ, USA) from cattle (*n* = 174; males = 34, females = 140) and buffaloes (*n* = 147; males = 39, females = 108), comprising 307 adults and 14 calves (Table 1). Samples were immediately stored at −20 °C. Genomic DNA was extracted from 100 µL aliquots of blood using the DNeasy Blood and Tissue Kit (Qiagen, Hilden, Germany), following the manufacturer’s protocol and DNA was preserved at −20 °C for downstream molecular analyses.

For the detection of *A. marginale*, a nested PCR assay targeting the *msp1β* gene was performed, using an established protocol [15]. The external primer pair AM456/AM1164 amplified a 700 bp fragment, while the internal primers AM100/AM101 yielded a 246 bp amplicon [15]. Purified genomic DNA was used as a template for the primary PCR whereas 10X diluted PCR amplicon was used in nested PCR. Thermal cycling conditions included an initial denaturation at 95 °C for 5 min, followed by 30 cycles of denaturation at 95 °C for 30 s, annealing at 60 °C for 30 s (61 °C for 10 s in the nested PCR), extension at 72 °C for 30 s, and a final extension at 72 °C for 5 min. Each reaction included known positive (genomic DNA from *A. marginale* positive bovine blood) and negative (Milli-Q water) controls. Aliquots (5 μL) of each PCR amplicon were detected by electrophoresis on GelRed-stained agarose gels (1.5% *w*/*v*) and scanned using a ChemiDoc MP imaging system (BioRad, Hercules, CA, USA).

To validate the results, 30% of PCR-positive amplicons were randomly selected from all districts for sequencing and subsequent analyses. Briefly, selected amplicons were purified using shrimp alkaline phosphatase and exonuclease I (Thermo Fisher Scientific, Waltham, MA, USA) as described by Werle et al. [17], followed by direct bi-directional Sanger sequencing. Following visual inspection of the forward and reverse reads representing each sequence in Geneious Prime 2025.0.3 (www.geneious.com), consensus sequences were obtained and conceptually translated to define open reading frames. All unique nucleotide sequences representing the complete sample set were deposited in GenBank.

Nucleotide sequences were aligned using Muscle5 [18] within Geneious Prime employing default settings, followed by calculation of pairwise nucleotide differences using BioEdit version 7.2.5 [19]. Subsequently, the unique sequences were compared with reference sequences in the National Centre for Biotechnology Information (NCBI) using BLASTn (https://blast.ncbi.nlm.nih.gov/Blast.cgi (accessed on 3 April 2025). Reference sequences of *A. marginale msp1β* reported previously from distinct geographical locations were retrieved from GenBank and aligned with the unique nucleotide sequences obtained in this study. Aligned sequences were trimmed to a uniform length of 216 nucleotides, and evolutionary models were established using MEGA 10.2.6 [20].

Using the best-fit evolutionary model, phylogenies were constructed using the Neighbour joining (NJ), Maximum Likelihood (ML), and Bayesian Inference (BI) methods in MEGA and Geneious [21,22,23]. The NJ and ML trees were constructed using 10,000 bootstrap replicates using the Kimura 2-parameter method and BI analysis was run for 10,000,000 generations (ngen = 10,000,000) to calculate posterior probabilities (pp), with every 2000th tree saved (samplefreq = 2000). Phylogenetic trees were rooted using *A. centrale* as an outgroup. Neutrality tests were conducted on *A. marginale* sequences (14 sequences, 216 bp) to assess deviations from the neutral theory of evolution. Tajima’s D was calculated using both MEGA X and DnaSP v5, while Fu’s Fs, Fu and Li’s D*, and Fu and Li’s F* statistics were computed using DnaSP [24]. Key parameters, including the number of segregating sites (S), nucleotide diversity (π), average pairwise, and nucleotide differences (k), were also determined. Statistical significance was assessed for Fu’s Fs and Fu and Li’s tests (*p* < 0.05).

Statistical analyses were conducted using Stata version 17.0 for Mac [25]. A *p*-value of <0.05 was considered statistically significant. Descriptive statistics were calculated for both outcome and explanatory variables. Categorical variables were summarised as frequencies and proportions. The apparent animal-level prevalence of *A. marginale* was calculated as the proportion of animals testing positive out of the total number of animals tested. A herd was classified as positive if ≥1 animal tested positive for *A. marginale*. The apparent herd-level prevalence was then calculated as the proportion of positive herds out of the total number of herds tested.

To determine the association of species, sex, age, and tick recovery with *A. marginale* positivity, a modified Poisson regression approach was employed to compute prevalence ratios (PR). This method was selected to avoid potential misinterpretation associated with inflated odds ratios from logistic regression due to the cross-sectional design and the relatively common nature of the outcome [26]. Consequently, a multilevel generalised linear mixed-effects model (GLMM) was developed using a robust variance–covariance estimator and incorporating district, village, and herd as random effects to account for the hierarchical structure of the data. All possible two-way interactions were explored to assess potential effect modification through a manual backward elimination process. Statistically significant interactions were retained in the final model [27]. The likelihood ratio test guided model selection, and the Akaike Information Criterion (AIC) was used to identify the best-fitting model. No effect modification was present. The final model is presented in Equation (1).

(1) $$\log\left(P_{{\left(AM\right)}_{ijkl}}\right)=\beta_{0}+\beta_{1}\cdot {\left(\mathrm{Species}\right)}_{l}+\beta_{2}\cdot {\left(\mathrm{Sex}\right)}_{l}+\beta_{3}\cdot {\left(\mathrm{Age}\right)}_{l}+\beta_{4}\cdot {\left(\mathrm{Tick}\;\mathrm{Recovery}\right)}_{l}+{Zu}_{ijk}$$

where

$P_{{\left(AM\right)}_{ijkl}}$ represents the probability of a positive nPCR result for *A. marginale* for the *l*th animal of the *k*th herd in the *j*th village within the *i*th district; $\beta_{0}$ is the intercept, which represents the log of the probability of a positive animal for *A. marginale* when all predictors are set to their reference categories; $\beta_{1}$ is the regression coefficient for species, which represents the difference in the log of the expected probability of *A. marginale* between cattle and buffaloes when all other predictors are held constant; $\beta_{2}$ is the regression coefficient for sex, which represents the difference in the log of the expected probability of *A. marginale* between male and female animals when all other predictors are held constant; $\beta_{3}$ is the regression coefficient for age, which represents the difference in log of the expected probability of *A. marginale* for adult animals compared to youngstock when all other predictors are held constant; $\beta_{4}$ is the regression coefficient for tick recovery, which represents the difference in log of the expected probability of *A. marginale* between animals with ticks and those without ticks when all other predictors are held constant; and ${Zu}_{ijk}$ represents the random intercepts for district, village, and herd, reflecting the nested hierarchical structure of the data. This accounts for within-cluster variation and helps capture the clustering effect across districts, villages, and herds. The final coefficients from Equation (1) were exponentiated to obtain PR along with 95% CI presented with respective *p*-values.

Finally, the prevalence of *A. marginale* was compared between different districts using a modified Poisson regression approach, as previously described. A multilevel GLMM was developed with *A. marginale* as a binary outcome and district as a categorical predictor while including village and herd as random effects. Potential confounding variables—age, sex, species, and tick recovery—were evaluated using a manual backward elimination process [27]. A covariate was considered a confounder if its removal changed the coefficient of at least one category of the main predictor (district) by 10% or more. Species and tick recovery were found to confound the association between district and *A. marginale* positivity and were thus retained in the final model, presented in Equation (2).

(2) $$\log\left(P_{{\left(AM\right)}_{ijkl}}\right)=\beta_{0}+\beta_{1}\cdot {\left(\mathrm{District}\right)}_{i}+\beta_{2}\cdot {\left(\mathrm{Species}\right)}_{l}+\beta_{3}\cdot {\left(\mathrm{Tick}\;\mathrm{Recovery}\right)}_{l}+{Zu}_{jk}$$

where

$\beta_{1}$ is the regression coefficient for a district, which represents the difference in the log of the expected probability of *A. marginale* for the given district compared to the reference district (Thatta) when all other predictors are held constant; $\beta_{2}$ and $\beta_{3}$ are the regression coefficient for confounders (species and tick recovery); and ${Zu}_{jk}$ represents the random intercepts for a village and herd, reflecting the nested hierarchical structure of the data for the given district. All subsequent analyses were conducted using the same statistical approach described above.

## 3. Results

Agarose gel electrophoresis of PCR amplicons revealed bands of expected sizes (primary PCR: 700 bp; nested PCR: 246 bp). Of 321 samples tested, 135 (42.1%) were test-positive (Table 1). The analysis revealed that the apparent animal-level prevalence of *A. marginale* was 42.1%, and the apparent herd-level prevalence was 58.0% (Table 1). The apparent animal-level prevalence was 62.5%, 19.6%, 44.6%, 61.3%, 54.6%, and 12.3% in Bahawalpur, Jhelum, Layyah, Okara, Sukkur, and Thatta, respectively (Table 1). Similarly, the apparent herd-level prevalence was 95.5%, 28.0%, 64.0%, 73.1%, 69.2%, and 23.1% in Bahawalpur, Jhelum, Layyah, Okara, Sukkur, and Thatta, respectively (Table 1). The prevalence of *A. marginale* was significantly higher in cattle (64.4%) than in buffaloes (15.6%) (χ2 = 75.63, *p* < 0.001). Females exhibited a slightly higher prevalence (43.5%) than males (37%), although the difference was not significant (χ2 = 0.75, *p* = 0.39).

The multi-level mixed effect modified Poisson regression model showed that the prevalence of *A. marginale* was significantly higher (PR = 3.69; 95% CI = 2.23–6.10) in cattle than in buffaloes (Table 2). Similarly, animals from which ticks were recovered were more frequently positive for *A. marginale* than those without ticks (PR = 1.39; 95% CI = 1.05–1.84)—tick data published elsewhere, please see: Ghafar et al. [11]. However, there was no difference between male and female animals (*p*-value = 0.635) and between young stock and adults (*p*-value = 0.113). Moreover, Bahawalpur (PR = 2.78; 95% CI = 1.51–5.11), Okara (PR = 3.08; 95% CI = 1.63–5.82), and Sukkur (PR = 3.26; 95% CI = 1.72–6.18) districts had a significantly higher prevalence than Thatta (Table 3), while the prevalence was not different in Jhelum (*p*-value = 0.705) and Layyah (*p*-value = 0.093) compared to Thatta.

Sequencing of 42 PCR amplicons of *A. marginale* resulted in 14 unique *msp1β* sequences (GenBank accession numbers: PV548909—PV548922). Nucleotide variability within the sequences of *msp1β* (aligned over 216 bp; GC content 51.9–54.2%) ranged from 0.5% to 12.1% (Supplementary Table S1). A total of 31 variations were found at 30 nucleotide positions, primarily attributed to transitions (A↔G and T↔C) at nucleotide positions 7, 13, 32, 34, 38, 48, 49, 51, 52, 62, 65, 82, 102, 174, 176, 193, 197, 202, 203, and 204. Additionally, transversions (A↔C, G↔C, G↔T) were also identified at nucleotide positions 26, 50, 52–54, 64, 175, 184, 195, 209, and 211. The protein alignment revealed multiple amino acid substitutions across 17 positions, with frequent changes at various positions (such as 3 [L→F], 5 [N→D], and 9 [D→A]) (Supplementary Figures S1 and S2).

Most sequences had a high similarity to those previously reported from Brazil (CP023730, CP023731, EU281852 (97.22–100%)), Thailand (MT96683, MT96684 (95.83–96.76%)), South Africa (KU647714 (98.14%)) and the USA (AF348137 (97.67%)). Neutrality tests for 14 unique sequences (216 bp) showed 30 polymorphic sites (S), with a nucleotide diversity (π) of 0.05647 and an average pairwise nucleotide difference (k) of 12.198. Theta (Θ) was estimated at 0.043674 per site and 9.748 per sequence. Tajima’s D statistic was positive (1.257271), for 14 unique sequences indicating an excess of intermediate-frequency alleles, although this result was not statistically significant (*p* > 0.10). Similarly, Fu and Li’s D* (0.69060) and F* (0.91452) were also positive, but not significant (*p* > 0.10). In contrast, Fu’s Fs statistic was significantly negative (−5.637, *p* = 0.004). Strobeck’s S statistic confirmed that all sequences were unique variants (S = 1.000 *p* = 0.004).

**Figure 2:**
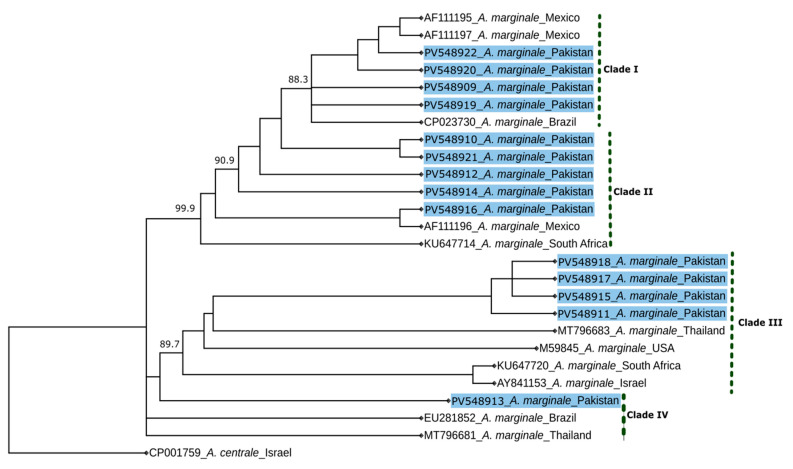
Genetic relationship of *msp1β* gene sequences of *Anaplasma* (*A*.) *marginale* identified in the present study (highlighted in blue: PV548909—PV548922) with reference sequences inferred using Bayesian Inference. Nodal support is given as a posterior probability value. The tree was rooted using *A. centrale* as an outgroup.

The molecular phylogenetic trees constructed using the NJ, ML, and BI methods had similar topologies; therefore, only the BI tree was selected to be presented here (Figure 2). The BI tree showed four distinct clades, with four of the sequences identified here (428, 203, 272, and 299) clustered with sequences from Mexico (AF111195 and AF111197) and Brazil (CP023730), supported by posterior probabilities of 88.3–94.8 (Figure 2). In clade II, five sequences (310, 5, 44, 363, and 464) from this study grouped with those from Mexico (AF111196) and South Africa (KU647714), with posterior probabilities ranging from 66.9 to 99.9 (Figure 2). Similarly, four sequences (198, 402, 460, and 126) formed a distinct clade with those with accession nos. MT796683 (Thailand), M59845 (USA), KU647720 (South Africa), and AY841153 (Israel), supported by probabilities ranging from 89.7 to 90.6 (Figure 2). Clade IV comprised a single sequence (39) grouped with accession nos. EU281852 (Brazil) and MT796681 (Thailand) (Figure 2). Sequences originating from water buffaloes grouped with sequences in clades I–III, while those from cattle grouped with sequences distributed across four clades (Figure 2).

## 4. Discussion

This comprehensive PCR-based study investigated the prevalence, and genetic diversity of *A. marginale* in clinically healthy cattle and buffalo populations across diverse agroecological zones of Pakistan. The findings contribute to our understanding of the epidemiology of bovine anaplasmosis with relevance to resource-poor smallholder farming communities.

This study found that the overall animal-level prevalence of *A. marginale* was 42.1%, with herd-level prevalence reaching 58.0%. Previous information on this important bovine pathogen from Pakistan is fragmented, with prevalences ranging from 2.7% to 53.7%, with most recent investigations reporting prevalences of *A. marginale* of 5.4% to 29.1% [8]. However, these previous studies were of limited regional significance as they were predominantly conducted in single districts (smaller administrative divisions within the province) [28,29,30,31,32,33]. Notably, no studies have systematically investigated this pathogen across Pakistan’s diverse agroecological zones. In the present study, the higher prevalences of *A. marginale* in bovines in Bahawalpur (62.5%) and Okara (61.3%) compared with that in the district of Thatta (12.3%) reflect agroecological variability. These differences could be attributed to differences in climatic conditions, tick densities, pathogen prevalence, tick species composition, and/or livestock farming practices [11]. This is particularly relevant to the distribution of tick vectors in these regions. Previous investigations have reported the presence of *Hyalomma anatolicum* as the main tick in arid regions, such as Bahawalpur, and reported the absence of *Rhipicephalus* (*R*.) *microplus* [11,34]. This is important since *R. microplus* is the primary vector for *A. marginale* globally, and the high prevalence of *A. marginale* in an area, despite the absence of a primary vector, suggests the presence of another active tick vector [3]. Recent investigations have reported the occurrence of *A. marginale* in *Hyalomma anatolicum*—collected from arid regions of Punjab province [10,34]. These findings indicate that *Hyalomma anatolicum* or *Hyalomma excavatum* might play an important role in transmitting *A. marginale* in regions where *R. microplus* is absent [35]. These findings are supported by recent evidence from a transmission study [36] in which intrastadial and transovarial transmissions of *A. marginale*, *T. annulata*, and lumpy skin disease virus were reported in male and female *Hyalomma anatolicum* ticks. A recent study of the prevalence of tick-borne pathogens in ticks reported that the highest prevalence of *A. marginale* in ticks was found in the district of Okara, this being attributed to a high prevalence of *R. microplus* in this region [10].

The significant disparity in prevalence between cattle (61.3%) and buffaloes (12.3%) is consistent with previous studies, suggesting lower susceptibility and higher natural resistance of water buffaloes to *A. marginale* infections [37,38,39,40]. The natural resistance in water buffaloes against tick-borne pathogens might partially be explained by their resistance to ticks, as evidenced by the lower larval survival rate of *R. microplus* on water buffaloes [11,38,41]. These findings are further supported by the association between tick presence and *A. marginale* infection (PR = 1.39; 95% CI = 1.05–1.84) reported here. In the present study, both water buffaloes and cattle were found positive for the same strains of *A. marginale*, suggesting that water buffaloes may serve as reservoir hosts, a role previously reported elsewhere and warranting further investigations [40]. The possible risk posed by subclinical infections becomes even more critical, considering Pakistan’s increasing importation of exotic cattle breeds, such as Holstein-Friesians and Jerseys, which are known to be highly susceptible to anaplasmosis and less adapted to local tick and pathogen pressures [8]. Without adequate screening and acclimatisation protocols, these genetically superior but immunologically naïve animals may experience severe clinical disease events upon exposure, jeopardising animal health, welfare, and productivity.

The higher prevalence of *A. marginale* in apparently healthy cattle (61.3%) compared with buffaloes might be an indicator of endemic stability [42,43] in cattle herds in this country. Endemic stability is a widely used term in relation to TBDs where, because of high-level exposure to a tick-borne pathogen at an early age (before nine months), the occurrence of clinical disease is very low, despite the presence of exposure and/or infection [42,43]. Calves are initially protected by maternal antibodies and later develop age-related resistance to clinical disease [42,43]. However, further investigation using serological tools and longitudinal surveillance studies is needed to confirm the proposed endemic stability status, since exposure varies across different times and geographical locations [42].

Genetic analyses revealed significant diversity in *msp1β* sequences, with 14 unique sequences identified. The high nucleotide diversity (π = 0.05647) could be indicative of a genetically dynamic population. The observed nucleotide variability (0.5–12.1%) aligns with global reports [44], reinforcing the evolutionary adaptability of *A. marginale* to different hosts and ecological pressures. Phylogenetic analysis clustered Pakistani samples into four distinct clades, revealing genetic linkages with samples characterised from Brazil, Mexico, South Africa, Thailand, and the USA. This clustering highlights the global distribution of *A. marginale*, potentially associated with historical livestock trade and migratory vectors. The clustering of some sequences (e.g., 39) with those from samples from Brazil and Thailand underscores the importance of investigating genetic relationships world-wide.

Despite the higher genetic heterogeneity found in our study, phylogenetic analysis of the *msp1β* sequences showed no distinct clustering based on host species (cattle vs. water buffaloes) or geographical locations. This finding suggests that *A. marginale* strains circulating in the study areas are potentially shared between multiple host species and across regions. It also suggests the likelihood of cross-species transmission of *A. marginale*, facilitated by mixed-species farming/overlapping grazing areas/similar husbandry practices and exposure to common tick vectors (i.e., *Hyalomma anatolicum* and *R. microplus*). In particular, the routine use of the same needles for vaccination, treatment, or other procedures across animals may also contribute to mechanical transmission of *A. marginale* between cattle and buffaloes. Similar findings have been reported in other endemic regions, where the same *A. marginale* genotypes were identified in both cattle and water buffaloes in Cuba, suggesting limited host-associated genetic structuring [38]. Nevertheless, larger-scale genome-wide studies would be needed to further resolve strain-level differences and confirm these transmission dynamics in different ecological contexts.

## 5. Conclusions

In conclusion, this study highlights the epidemiological and genetic complexity of *A. marginale* and possible risks of production losses associated with sub-clinical infection in resource-poor small-holder farming systems in developing countries such as Pakistan. The present findings contribute to an understanding of the epidemiology of *A. marginale* and provide insights that will contribute to the improved control of bovine anaplasmosis. Future research should focus on (i) identifying the tick species responsible for the transmission of *A. marginale* in Pakistan, (ii) conducting longitudinal studies to elucidate seasonal dynamics, and (iii) gaining deeper insight into the genetic diversity of *A. marginale* via advanced genomic sequencing. Serological surveys combined with the use of such advanced molecular tools should provide an enhanced understanding of host immune responses and infection intensities, aimed at supporting the design of region-specific intervention strategies.

## Figures and Tables

**Table 1:** Demographic distribution and apparent prevalence of *Anaplasma marginale* (positive in nested PCR) in blood samples collected from cattle and buffaloes across two provinces and six districts of Pakistan.

Province	District	Positive/Tested Herds No.	Positive/Tested Animals No.					
			Age Groups		Cattle		Buffalo	
			Adult	Calf	Female	Male	Female	Male
Punjab	Bahawalpur	21/22	25/40	-	17/21	5/5	3/11	0/3
	Jhelum	7/25	10/47	0/4	10/22	0/4	0/18	0/7
	Layyah	16/25	25/53	0/3	23/39	1/5	1/9	0/3
	Okara	19/26	36/56	2/6	21/28	7/7	9/23	1/4
Sindh	Sukkur	18/26	30/55	-	17/19	7/8	3/19	3/9
	Thatta	6/26	7/56	0/1	2/11	2/5	2/28	1/13
Total		87/150	133/307	2/14	90/140	22/34	18/108	5/39

**Table 2:** Prevalence ratios (PR) and 95% CI from the multi-level modified Poisson regression model assessing the association of age, sex, species, and tick recovery with a positive nested PCR result for *A. marginale* (n = 321) in six districts of Punjab and Sindh, Pakistan.

Variable	Category	Animals No. (%)	Prevalence Ratio (95% CI)	*p*-Value
Age	Young stock	14 (4.4)	Referent	
	Adults	307 (95.6)	2.78 (0.79–9.82)	0.113
Sex	Male	73 (22.7)	Referent	
	Female	248 (77.3)	0.95 (0.77–1.17)	0.635
Species	Buffaloes	147 (45.8)	Referent	
	Cattle	174 (54.2)	3.69 (2.23–6.10)	<0.001
Ticks Recovery	Ticks absent	201 (62.6)	Referent	
	Ticks present	120 (37.4)	1.39 (1.05–1.84)	0.021

**Table 3:** Adjusted prevalence ratios (PR) and 95% CI from the multi-level modified Poisson regression model, adjusted for species and tick recovery, assessing the association of district with a positive nPCR results for *A. marginale* (n = 321) in Punjab and Sindh, Pakistan.

District	Animals No. (%)	Prevalence Ratio (95% CI)	*p*-Value
Thatta	57 (17.8)	Referent	
Bahawalpur	40 (12.5)	2.78 (1.51–5.11)	0.001
Jehlum	51 (15.9)	1.17 (0.53–2.57)	0.705
Layyah	56 (17.5)	1.93 (0.90–4.16)	0.093
Okara	62 (19.3)	3.08 (1.63–5.82)	0.001
Sukkur	55 (17.1)	3.26 (1.72–6.18)	<0.001

## Data Availability

The unique nucleotide sequences generated in this study are available in the NCBI GenBank database under accession numbers PV548909–PV548922.

## Supplementary Materials

The following supporting information can be downloaded at: https://www.mdpi.com/article/10.3390/pathogens14050499/s1, Figure S1: Nucleotide alignment of partial *msp1β* gene sequences of *Anaplasma marginale* identified in this study. Dots (.) represent nucleotides identical to the top sequence, and letters indicate nucleotide substitutions. The alignment illustrates sequence variation among the 14 unique variants; Figure S2: Amino acid alignment of partial *msp1β* sequences of *Anaplasma marginale* identified in this study. Dots (.) indicate residues identical to the top sequence; letters indicate amino acid substitutions. Variations highlight the genetic heterogeneity among the 14 unique sequence types; Table S1: Pairwise comparison of *msp1β* nucleotide sequences (aligned over 216 bp) of *Anaplasma marginale* determined herein. Nucleotide similarity and percentage differences are given above and below the diagonal, respectively.
