# Supplementary material for: High Prevalence and Genetic Heterogeneity of Anaplasma marginale in Smallholder Bovine Populations of Pakistan, and Its Implications
Source: Pathogens. 2025 May 20;14(5):499. doi: 10.3390/pathogens14050499 (PMC12114811; doi:10.3390/pathogens14050499)
Supplement: Supplementary file 1 [file pathogens-14-00499-s001.zip › pathogens-3644472-supplementary.pdf]

```
          ....|....| ....|....| ....|....| ....|....| ....|....| ....|....| ....|....|
                  10         20         30         40         50         60         70
PV548909  CTACCGCTCA TGAATGAAGC ACCTGACACT GATGAGCGGG TTGTTGTGCA GTATGGTGAG GAGAGAGAAT
PV548910  .......... .......... .......... .......... .......... .......... ..........
PV548911  ......T... ..G....... .....C.... .......A.. .......ATC AGCG...... ...CA.....
PV548912  .......... .......... .......... .......... .......... .......... ..........
PV548913  ......T... ..G....... .....C.... .......A.. .......ATC AGCG...... ...CA.....
PV548914  .......... .......... .......... .......... .......... .......... ..........
PV548915  .......... ..G....... .....C.... .......A.. .......ATC AGCG...... .G.CA.....
PV548916  ......T... ..G....... .......... .G........ .......... .C........ ..........
PV548917  .......... .......... .....C.... .......A.. .......ATC AGCG...... ...CA.....
PV548918  .......... ..G....... .....C.... .......A.. .......ATC AGCG...... ...CA.....
PV548919  .......... .......... .......... ...A...... .......... .......... ..........
PV548920  .......... .......... .......... .G........ .......... .......... ..........
PV548921  .......... .......... .......... .......... .......... .......... ..........
PV548922  .......... ..G....... .......... .G........ .......... .......... ..........

          ....|....| ....|....| ....|....| ....|....| ....|....| ....|....| ....|....|
                  80         90        100        110        120        130        140
PV548909  TTGGCAAGGC AGCAGCCTGG GGTCTAGCAG GTTTCAAGCG TACAGTGGAT GAAAGCCTGG AGATGTTAGA
PV548910  .......... .......... .......... .......... .......... .......... ..........
PV548911  .......... .......... .......... .......... .......... .......... ..........
PV548912  .......... .......... .......... .......... .......... .......... ..........
PV548913  .......... ......T... .......... .C........ .......... .......... ..........
PV548914  .......... ......T... .......... .......... .......... .......... ..........
PV548915  .......... .......... .......... .......... .......... .......... ..........
PV548916  .......... .......... .......... .......... .......... .......... ..........
PV548917  .......... .......... .......... .......... .......... .......... ..........
PV548918  .......... .......... .......... .......... .......... .......... ..........
PV548919  .......... .......... .......... .......... .......... .......... ..........
PV548920  .......... .......... .......... .......... .......... .......... ..........
PV548921  .......... .......... .......... .......... .......... .......... ..........
PV548922  .......... .......... .......... .......... .......... .......... ..........

          ....|....| ....|....| ....|....| ....|....| ....|....| ....|....| ....|....|
                 150        160        170        180        190        200        210
PV548909  CCGAGGCATG CACATGCTCG CGGAAGGCCA GGCGAGGATA TCAGAGGGGA TTAACGCCAA GGATACTGCA
PV548910  .......... .......... .......... ...ACA.... .......... .......... ..........
PV548911  .......... .......... .......... .......... .......... .......... ..........
PV548912  .......... .......... .......... .......... .......... ..G.A..... .AG.....A.
PV548913  .......... .......... .......... ...ACA.... ...C...... ..G.A..... .AG.....A.
PV548914  .......... .......... .......... ...ACA.... ...C...... ..G.A..... .AG.....A.
PV548915  .......... .......... .......... .......... .......... .......... ..........
PV548916  .......... .......... .......... ...ACA.... ...C...... ..G.A.T... .AGC......
PV548917  .......... .......... .......... .......... .......... .......... ..........
PV548918  .......... .......... .......... .......... .......... .......... ..........
PV548919  .......... .......... .......... .......... .......... .......... ..........
PV548920  .......... .......... .......... .......... .......... .......... ..........
PV548921  .......... .......... .......... ...AC..... .......... .......... ..........
PV548922  .......... .......... .......... .......... .......... .......... ..........

          ....|.

PV548909  ATAGTT
PV548910  C.....
PV548911  ......
PV548912  C.....
PV548913  C.....
PV548914  C.....
PV548915  ......
PV548916  C.....
PV548917  ......
PV548918  ......
PV548919  ......
PV548920  ......
PV548921  C.....
PV548922  ......
```

Figure S1: Nucleotide alignment of partial p*msp1β* gene sequences of *Anaplasma marginale* identified in this study. Dots (.) represent nucleotides identical to the top sequence, and letters indicate nucleotide substitutions. The alignment illustrates sequence variation among the 14 unique variants.

```
            ....|....| ....|....| ....|....| ....|....| ....|....| ....|....| ....|....| ..
                    10         20         30         40         50         60         70
PV548909    LPLMNEAPDT DERVVVQYGE EREFGKAAAW GLAGFKRTVD ESLEMLDRGM HMLAEGQARI SEGINAKDTA IV
PV548910    .......... .......... .......... .......... .......... ........Q. .......... L.
PV548911    ..F.D...A. ..Q...SA.. .Q........ .......... .......... .......... .......... ..
PV548912    .......... .......... .......... .......... .......... .......... ....E..S.E L.
PV548913    ..F.D...A. ..Q...SA.. .Q........ .......... .......... ........Q. .Q..E..S.E L.
PV548914    .......... .......... .......... .......... .......... ........Q. .Q..E..S.E L.
PV548915    ....D...A. ..Q...SA.. GQ........ .......... .......... .......... .......... ..
PV548916    ..F.D..... G.....H... .......... .......... .......... ........Q. .Q..EV.S.. L.
PV548917    ........A. ..Q...SA.. .Q........ .......... .......... .......... .......... ..
PV548918    ....D...A. ..Q...SA.. .Q........ .......... .......... .......... .......... ..
PV548919    .......... .K........ .......... .......... .......... .......... .......... ..
PV548920    .......... G......... .......... .......... .......... .......... .......... ..
PV548921    .......... .......... .......... .......... .......... .......... .......... L.
PV548922    ....D..... G......... .......... .......... .......... .......... .......... ..
```

Figure S2: Amino acid alignment of partial p*msp1β* sequences of *Anaplasma marginale* identified in this study. Dots (.) indicate residues identical to the top sequence; letters indicate amino acid substitutions. Variations highlight the genetic heterogeneity among the 14 unique sequence types.

Table S1. Pairwise comparison of *msp1β* nucleotide sequences (aligned over 216 bp) of *Anaplasma marginale* determined herein. Nucleotide similarity and percentage differences are given above and below the diagonal, respectively

**Ids**	**272**	**310**	**126**	**44**	**39**	**363**	**460**	**464**	**402**	**198**	**299**	**203**	**5**	**428**
**272**		0.981	0.939	0.972	0.884	0.949	0.939	0.93	0.949	0.944	0.995	0.995	0.986	0.99
**310**	1.9		0.921	0.962	0.902	0.967	0.921	0.949	0.93	0.925	0.976	0.976	0.995	0.972
**126**	6.1	7.9		0.912	0.944	0.888	0.99	0.893	0.99	0.995	0.935	0.935	0.925	0.939
**44**	2.8	3.8	8.8		0.912	0.976	0.912	0.949	0.921	0.916	0.967	0.967	0.967	0.962
**39**	11.6	9.8	5.6	8.8		0.935	0.935	0.921	0.935	0.939	0.879	0.879	0.898	0.884
**363**	5.1	3.3	11.2	2.4	6.5		0.888	0.962	0.898	0.893	0.944	0.944	0.962	0.939
**460**	6.1	7.9	1	8.8	6.5	11.2		0.884	0.99	0.995	0.935	0.935	0.925	0.939
**464**	7	5.1	10.7	5.1	7.9	3.8	11.6		0.884	0.888	0.925	0.935	0.944	0.939
**402**	5.1	7	1	7.9	6.5	10.2	1	11.6		0.995	0.944	0.944	0.935	0.939
**198**	5.6	7.5	0.5	8.4	6.1	10.7	0.5	11.2	0.5		0.939	0.939	0.93	0.944
**299**	0.5	2.4	6.5	3.3	12.1	5.6	6.5	7.5	5.6	6.1		0.99	0.981	0.986
**203**	0.5	2.4	6.5	3.3	12.1	5.6	6.5	6.5	5.6	6.1	1		0.981	0.995
**5**	1.4	0.5	7.5	3.3	10.2	3.8	7.5	5.6	6.5	7	1.9	1.9		0.976
**428**	1	2.8	6.1	3.8	11.6	6.1	6.1	6.1	6.1	5.6	1.4	0.5	2.4	
